# Correlation between bone mineral density and sarcopenia in US adults: a population-based study

**DOI:** 10.1186/s13018-023-04034-7

**Published:** 2023-08-09

**Authors:** Lulu Cheng, Siyu Wang

**Affiliations:** 1 College of Acupuncture-Moxibustion and Tuina, Anhui University of Chinese Medicine, Hefei, 230012 China; 2 Graduate School, Wuhan Sports University, Wuhan, 430079 China

**Keywords:** Lumbar bone mineral density, Osteoporosis, Sarcopenia, Muscle–bone–lipid, NHANES

## Abstract

**Introduction:**

In the aging process of the body, in addition to changes in fat and muscle content, there is also bone loss, implying the possibility of a strong muscle–bone–lipid link. In this study, we initially investigated the relationship between lumbar BMD and low muscle mass and the relationship between “muscle–bone–lipid.”

**Methods:**

The datasets from the National Health and Nutrition Examination Survey (NHANES) 2011–2018 were used in a cross-sectional investigation. BMD and appendicular skeletal muscle (ASM) were measured by dual-energy X-ray absorptiometry (DXA), and appendicular skeletal muscle was adjusted by body mass index (BMI) as a marker of sarcopenia. Weighted multivariate regression and logistic regression analysis were used to explore the independent relationship between lumbar BMD and sarcopenia. Fitted smoothing curves and threshold effect analysis were used to describe the nonlinear relationship.

**Result:**

In 8386 participants with ages 20–59 years, there was a negative association between lumbar BMD and sarcopenia. In the fully adjusted model, the risk of developing sarcopenia decreased by 93% for each 1-unit increase in lumbar BMD (OR = 0.07, 95%CI 0.03–0.20). The risk of sarcopenia was 58% lower in participants in the highest quartile of lumbar BMD than in those in the lowest quartile (OR = 0.42, 95%CI 0.27–0.64). This negative association was more pronounced in the population of women with BMI ≥ 25.

**Conclusion:**

Our findings suggest that lumbar BMD is negatively associated with sarcopenia in US adults. The dynamic balance between “muscle–bone–lipid” is likely to be related to the pathogenesis of bone loss.

**Supplementary Information:**

The online version contains supplementary material available at 10.1186/s13018-023-04034-7.

## Background

With the increasingly prominent global aging problem, sarcopenia, osteoporosis (OP), and other aging-related diseases are gradually becoming a research hotspot in the field of geriatrics [1]. There is a strong association between sarcopenia and OP. Muscular and skeletal disorders often appear simultaneously and interact with each other in the elderly population, the harm and impact of which significantly increases the individual and socioeconomic burden [2]. In addition, the prevalence of sarcopenic obesity in the elderly is increasing due to increased aging. As age increases, the body’s muscle mass and function decrease and adipose tissue accumulates. Its overall expression is a decrease in body muscle quality, an increase in adiposity, an increase in inflammatory factors, and a decrease in activity [3, 4].

As a chronic disease of the elderly, OP has a slow and insidious onset. It is known as the “silent killer” in academic circles. As the body ages, physiological changes in muscle, bone, and lipid are more apparent in the phenotype [5]. In postmenopausal women, bone mass decreases more rapidly than in men, at a rate of 1–2% per year. In contrast, unlike the decrease in muscle mass and bone tissue, adipose tissue may plateau or decline during advanced age. All of these changes can lead to a decrease in overall strength and function of the individual, increasing the risk of falls and fractures [6]. With the increase in age, in addition to the changes of fat and muscle content, there is also bone loss, implying the possibility of a strong “muscle–bone–lipid” link. There are few studies on the relationship between sarcopenia and the development of lumbar BMD. The current “gold standard” for the diagnosis of OP is dual-energy X-ray absorptiometry (DXA). This technique is easy to use and has high accuracy and precision [7]. DXA can also be used as a standard method for body composition measurement to obtain muscle, lipid, and bone mineral contents and their ratios in the whole body, as well as in the trunk and extremities. In this review, a population-based cross-sectional study was conducted among adult participants of the National Health and Nutrition Examination Survey (NHANES) to investigate the correlation between lumbar BMD and sarcopenia.

## Material and methods

### Study population

An ongoing nationwide, population-based survey of nutrition and health in the USA is the NHANES database. It employs sophisticated probability sampling methods as opposed to a straightforward random sample drawn from the population of the USA. Visit (https://www.cdc.gov/nchs/nhanes/index.htm) to learn more about the statistics. The website provides information on the continuous design of the NHANES survey, the informed consent forms that all study participants signed, and the fact that the National Center for Health Statistics Ethics Review Board approved all study protocols prior to data collection. We used publicly accessible data from four NHANES two-year cycles (2011–2012, 2013–2014, 2015–2016, and 2017–2018) for analysis in this study.

**Fig. 1:**
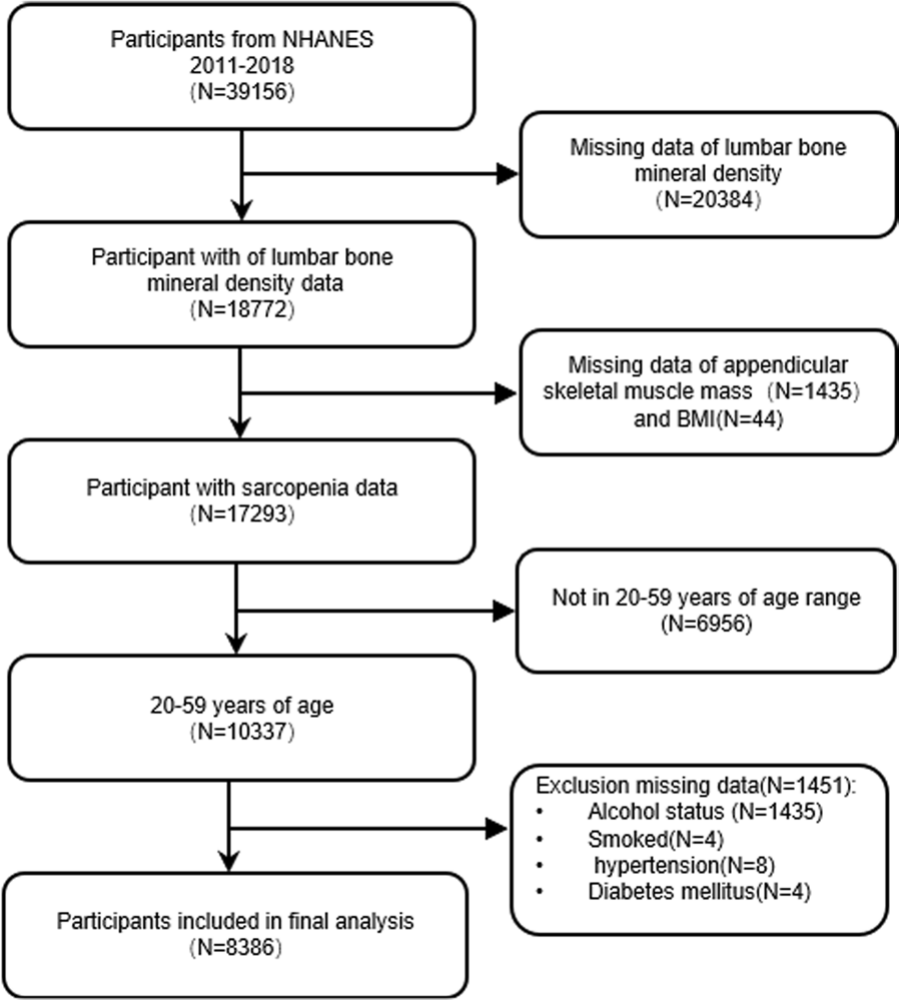
Flowchart of participant selection. NHANES, National Health and Nutrition Examination Survey

Among the 39,156 participants, we excluded 20,384 participants without lumbar BMD data, 1479 participants without reliable DXA and body mass index (BMI) data, 6,956 underage participants, and 1451 remaining participants with missing covariates. Lastly, 8,386 participants were involved (Fig. 1).

### Study variables

Participants aged 8–59 underwent DXA whole-body scans using Hologic Discovery model A densitometers (Hologic, Bedford, Massachusetts, USA). Participants who were pregnant, obese (> 136 kg), tall (> 196 cm), and taking radiographic contrast (barium) within the last 7 days were unable to participate in DXA. Dual-energy X-ray absorptiometry was performed by a qualified radiology technician using Hologic QDR 4500A equipment and Apex software version 3.2 to assess lumbar BMD.

DXA was used to calculate appendicular skeletal muscle mass (ASM), which is the total lean mass of the arms and legs. A detailed description of DXA measurement data gathering and accuracy can be found elsewhere (https://www.cdc.gov/nchs/nhanes/). In this study, sarcopenia index was calculated as ASM adjusted by BMI (ASM/BMI), and males were classified as having sarcopenia if sarcopenia index < 0.789 and females < 0.512, based on the criteria designated by a recent consensus meeting “Foundation for the National Institutes of Health (FNIH) Sarcopenia Project” and widely used in recent research.

Covariates in multivariate models may lead to confounding correlations between lumbar BMD and sarcopenia. Age, sex, race, education level, marital status, BMI, income to poverty ratio(PIR), smoked at least 100 cigarettes during the lifespan of data collection, alcohol drinking status, diabetes status, hypertension status, creatinine, serum uric acid, glycohemoglobin, fasting glucose, high-density lipoprotein cholesterol(HDL-C), total cholesterol, triglycerides, low-density lipoprotein cholesterol(LDL-C), and albumin were all covariates.

### Statistical analysis

To study the relationship between lumbar BMD and sarcopenia, a weighted multivariate logistic regression model was used. To calculate the difference between each group, we utilized the weighted test for categorical variables and the weighted linear regression model for continuous variables. The stratified multivariate regression analysis was used for the subgroup analysis. Smooth curve fits and generalized additive models were used to investigate the nonlinear relationship between lumbar BMD and Sarcopenia.

R studio (version 4.2.2) and EmpowerStats (version 4.1) were used for statistical analysis. We found that *P* value of 0.05 was significant, and we also utilized a weighting approach to reduce major swings in the dataset.

## Results

### Baseline characteristics of participants

Our study included a total of 8386 participants, 53.06% of whom were male, with a mean age of 39.21 ± 11.75. The mean lumbar BMD level was 1.04 ± 0.15, of whom 6.69% had Sarcopenia. Compared to participants without sarcopenia, participants with sarcopenia were older, more Mexican American, and less Non-Hispanic white, adults with sarcopenia had higher BMI, lower PIR, and lower education level. In addition, gender, marital status, alcohol drinking status, smoking status, hypertension, diabetes, uric acid, glycohemoglobin, fasting glucose, HDL-C, total cholesterol, triglycerides, LDL-C, and albumin were significantly different between the two groups. The weighted demographic baseline characteristics of the included participants are shown in Table 1.

**Table 1:** Weighted characteristics of the study population based on sarcopenia

	Non-Sarcopenia (N = 7739)	Sarcopenia (N = 647)	*P* value
Age (years)	38.94 ± 11.69	42.99 ± 11.88	< 0.0001
Sex (%)			< 0.0001
Male	52.44	61.62	
Female	47.56	38.38	
Race (%)			< 0.0001
Mexican American	9.02	24.10	
Other Hispanic	6.75	11.32	
Non-Hispanic white	64.06	53.09	
Non-Hispanic black	11.49	3.32	
Other race	8.68	8.17	
Education level (%)			< 0.0001
Less than 9th grade	2.66	8.37	
9th–11th grade	8.72	12.58	
High school graduate/GED or equivalent	21.04	26.70	
Some college or AA degree	33.47	33.01	
College graduate or above	34.10	19.35	
Marital, N(%)			< 0.0001
Married/living with partner	61.81	60.88	
Separated/Divorced/Widowed	12.65	18.66	
Never married	25.54	20.46	
Body mass index(kg/m^2^)			< 0.0001
< 25	33.63	5.70	
25–29.9	34.11	20.05	
≥ 30	32.26	74.25	
Alcohol drinking status			0.0011
Yes	14.87	20.01	
No	85.13	79.99	
Smoked ≥ 100 cigarettes in life (%)			0.0292
Yes	44.00	48.74	
No	56.00	51.26	
Hypertension (%)			< 0.0001
Yes	21.51	31.83	
No	78.49	68.17	
Diabetes (%)			< 0.001
Yes	4.83	15.01	
No	95.17	84.99	
Creatinine (mg/dL, mean ± SD)	125.96 ± 84.10	127.25 ± 77.64	0.7245
Uric acid (umol/L, mean ± SD)	5.33 ± 1.34	5.84 ± 1.38	< 0.0001
Glycohemoglobin (%)	5.47 ± 0.84	5.87 ± 1.17	< 0.0001
Fasting glucose (mg/dL, mean ± SD)	103.53 ± 28.90	115.83 ± 41.86	< 0.0001
HDL-C(mg/dL, mean ± SD)	53.29 ± 15.83	47.93 ± 13.53	< 0.0001
Total cholesterol(mg/dL, mean ± SD)	191.43 ± 40.26	197.31 ± 42.75	0.0012
Triglyceride(mg/dL, mean ± SD)	119.13 ± 105.67	150.42 ± 100.35	< 0.0001
LDL-C(mg/dL, mean ± SD)	113.12 ± 33.85	121.33 ± 36.24	0.0002
Albumin (g/dL, mean ± SD)	4.34 ± 0.33	4.21 ± 0.34	< 0.0001
Income to poverty ratio	3.06 ± 1.66	2.57 ± 1.63	< 0.0001
Lumbar BMD (g/cm^2^, mean ± SD)	1.04 ± 0.15	0.97 ± 0.14	< 0.0001

Mean ± SD for continuous variables: the p value was calculated by a weighted linear regression model. % for categorical variables: the p value was calculated by a weighted chi-square test. HDL-C, high-density lipoprotein cholesterol; LDL-C, low-density lipoprotein cholesterol; BMD, bone mineral density

### Association between lumbar BMD and sarcopenia

**Fig. 2:**
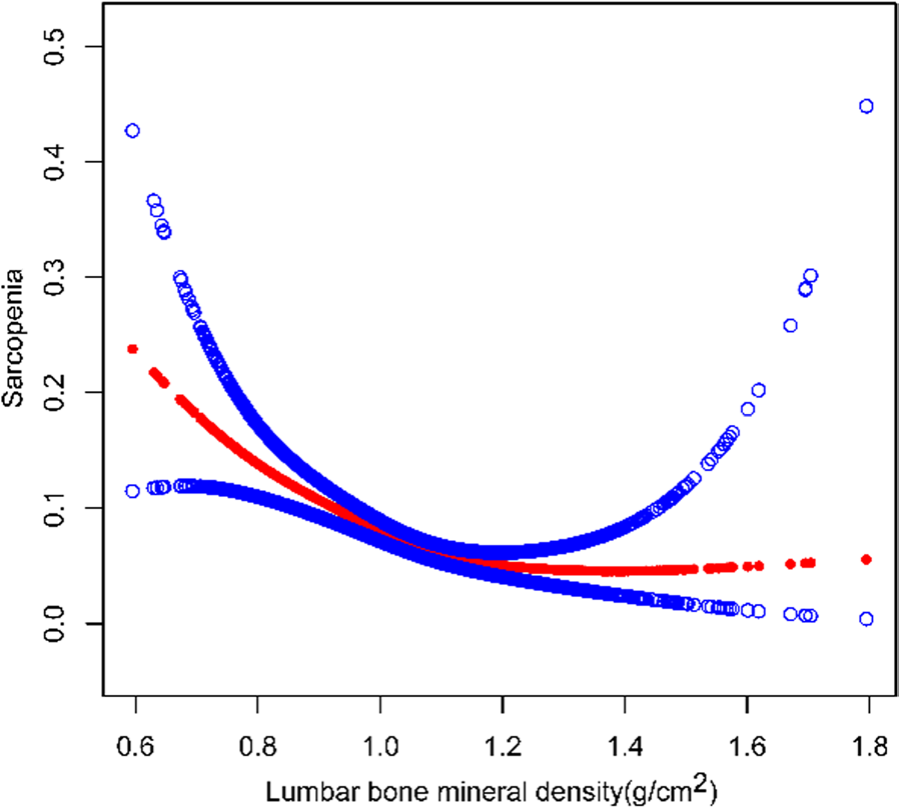
The association between lumbar BMD and sarcopenia. The solid red line represents the smooth curve fit between variables. Blue bands represent the 95% confidence interval from the fit

Multiple regression analysis showed that lumbar BMD was strongly and independently negatively associated with sarcopenia in all models. When adjusted for the main demographic variables (model 2), the risk of sarcopenia decreased by 94% for every 1 g/cm^2^ increase in BMD (OR = 0.06, 95%CI 0.03–0.11). After adjusting for covariates, our findings suggest that for every unit increase in BMD, the risk of sarcopenia is reduced by 93%. Similarly, sensitivity analysis by lumbar spine bone density quartiles showed a corrected OR (reference Q1) of 0.42 (95% CI 0.28–0.64; *P* < 0.01) for Q4 in both the unadjusted and fully adjusted models, and the trend test between them remained significant (*P* for trend < 0.0001). This suggests a stable negative correlation between increased BMD and the risk of sarcopenia, which is statistically significant (Table 2). When stratified by sex, age, and BMI, a negative association between lumbar BMD and sarcopenia was found. In addition, we further performed smooth curve fitting and generalized additive models for defining the nonlinear association between lumbar BMD and sarcopenia. The results validated the negative nonlinear relationship between lumbar BMD and sarcopenia (Fig. 2, Additional file 1).

**Table 2:** The association between lumbar BMD and Sarcopenia

	Model1 [OR (95% CI)]	Model2 [OR (95% CI)]	Model3 [OR (95% CI)]
Lumbar BMD	0.02(0.01,0.03)	0.06(0.03,0.11)	0.07(0.02,0.19)
Lumbar BMD (continuous)			
Q1	Reference	Reference	Reference
Q2	0.58(0.47,0.71)	0.67(0.55,0.83)	0.79(0.57,1.09)
Q3	0.35(0.28,0.44)	0.47(0.37,0.59)	0.39(0.26,0.59)
Q4	0.24(0.19,0.31)	0.36(0.28,0.48)	0.42(0.28,0.64)
*P* for trend	< 0.0001	< 0.0001	< 0.0001
Stratified by gender			
Male	0.04(0.02,0.08)	0.09(0.04,0.21)	0.14(0.03,0.53)
Female	0.01(0.00,0.02)	0.04(0.01,0.10)	0.04(0.01,0.21)
Stratified by BMI			
< 25	0.01(0.00,0.05)	0.03(0.00,0.29)	0.31(0.01,8.07)
25–29.9	0.01(0.00,0.02)	0.02(0.01,0.08)	0.01(0.00,0.07)
≥ 30	0.03(0.02,0.07)	0.09(0.04,0.20)	0.11(0.03,0.40)
Stratified by age			
< 30	0.01(0.00,0.03)	0.01(0.00,0.08)	0.02(0.00,0.52)
30–40	0.01(0.00,0.05)	0.02(0.01,0.12)	0.01(0.00,0.16)
40–50	0.01(0.00,0.05)	0.03(0.01,0.12)	0.03(0.00,0.26)
≥ 50	0.01(0.04,0.24)	0.20(0.08,0.50)	0.21(0.05,0.98)

Model 1: no covariates were adjusted

Model 2: age, sex, and race were adjusted

Model 3: age, sex, race, education level, marital status, smoking status, alcohol status, diabetes status, hypertension status, creatinine, serum uric acid, glycohemoglobin, fasting glucose, HDL-C, total cholesterol, triglycerides, LDL-C, PIR, and albumin were adjusted. 95% CI, 95% confidence interval; OR, odds ratio; Q, quartile; BMI, body mass index

In the subgroup analysis stratified by gender, BMI, and race, the model is not adjusted for sex, BMI, and race, respectively

### Subgroup analysis

We conducted the subgroup analysis stratified by gender, age, BMI, hypertension, diabetes, smoking status, alcohol status, total cholesterol, triglycerides, LDL-C, and HDL-C to further explore the association of lumbar BMD with sarcopenia in different population settings by stratified weighted multivariate regression analysis and tested the interactions (Table 3). Regarding the correlation between lumbar BMD and sarcopenia, interaction tests showed a significant interaction between lumbar BMD and sarcopenia for alcohol status (P = 0.0391), but gender, BMI, age, smoking status, hypertension, diabetes, smoking status, alcohol status, total cholesterol, triglycerides, LDL-C, and HDL-C had no significant effect on this negative correlation (P for interaction > 0.05). These results indicated that the negative association between lumbar BMD and sarcopenia was similar in the population with gender, age, BMI, hypertension status, diabetes status, and smoking status and could also be appropriate for the participants with dyslipidemia.

**Table 3:** Subgroup analysis for the association between lumbar BMD and sarcopenia

Subgroup	Sarcopenia [OR(95%CI)]	*P* for interaction
Sex		0.0730
Man	0.15(0.04,0.56)	
Female	0.02(0.00,0.11)	
BMI		0.0594
< 25	0.29(0.01,5.84)	
25–29.9	0.01(0.00,0.07)	
≥ 30	0.12(0.03,0.44)	
Year		0.1438
< 30	0.02(0.00,0.51)	
30–39	0.01(0.00,0.18)	
40–49	0.04(0.01,0.30)	
≥ 50	0.25(0.06,1.12)	
Hypertension		0.0647
Yes	0.25(0.05,1.24)	
No	0.04(0.01,0.13)	
Diabetes		0.1011
Yes	0.48(0.03,6.92)	
No	0.04(0.01,0.13)	
Smoking status		0.1287
Yes	0.15(0.04,0.62)	
No	0.03(0.01,0.13)	
Alcohol status		0.0391
Yes	0.61(0.06,5.86)	
No	0.04(0.01,0.13)	
Total cholesterol		0.8709
< 200	0.07(0.02,0.28)	
≥ 200	0.06(0.01,0.27)	
Triglycerides		0.2159
< 150	0.04(0.01,0.15)	
≥ 150	0.17(0.03,1.06)	
HDL-C		0.7099
< 40	0.04(0.00,0.38)	
≥ 40	0.07(0.02,0.21)	
LDL-C		0.5268
< 130	0.05(0.01,0.19)	
≥ 130	0.10(0.02,0.49)	

The results of subgroup analysis were adjusted for all covariates except effect modifier. BMI, body mass index; HDL-C, high-density lipoprotein cholesterol; LDL-C, low-density lipoprotein cholesterol

## Discussion

In this nationally representative study, we investigated the relationship between lumbar spine bone density and sarcopenia and found a negative association between lumbar BMD and sarcopenia. Furthermore, this association remained significant after adjusting for common influences between lumbar BMD and sarcopenia. Considering the large sample and reasonable quality control, our analysis should be reliable. In particular, in the analysis by gender, age, and BMI subgroups, we found the strongest and most significant negative association effect between lumbar BMD and sarcopenia when BMI ≥ 25 among participants of different age groups.

During the aging process, muscle, bone, and lipid content change to varying degrees. As age increases, bone metabolism is in a state of “low conversion” and bone microstructure is significantly damaged. The prevalence of sarcopenia increases from 13 to 50% between the ages of 60 and 80 years, increasing the risk of fragility fractures [8, 9]. It has been shown that human muscle mass peaks between 30 and 40 years of age and then gradually decreases [10]. Muscle mass decreases by 0.45% per year in men and 0.37% per year in women. Age-related decline in muscle strength is an important predictor of functional activity impairment, falls, fractures, and death in the elderly [11–13]. In the elderly population, loss of bone tissue leads to deterioration of skeletal microarchitecture and increased fracture risk. Its increase in the number and activity of osteoclasts at the cellular level disrupts the connectivity of bone trabeculae and increases cortical porosity. Reduced bone density and bone mass, in turn, decrease the mechanical load-bearing properties of the bone and increase the susceptibility to fracture [14]. Also, studies [15, 16] have shown that OP greatly increases the risk of sarcopenia, which may be related to the fact that the decrease in bone mass with age alters the biomechanical characteristics of muscles and affects their morphology and function. A British study [17] reported a prevalence of sarcopenia of up to 50% in postmenopausal women with OP. Di Monaco et al. [18] performed DXA scans in 340 Italian Caucasian female patients with hip fractures and found an OR for T values ≤ − 2.5 in women with sarcopenia of 1.80 (95% CI 1.07, 3.02). The association of sarcopenia with OP was demonstrated. This is in agreement with the results of the present study. In addition, during our correction for confounding factors in the interaction test for lumbar spine bone density and sarcopenia, we also found a more significant effect of gender on the negative association between lumbar spine bone density and sarcopenia. This may be related to the fact that estrogens, androgens, and testosterone are all associated with the metabolism of skeletal and muscular tissues in humans [19]. Estrogens maintain skeletal muscle contractility and prevent OP [20], while androgens and testosterone promote myoblast proliferation [21]. And as age increases, the function of endocrine organs decreases, which also leads to a decrease in muscle mass and a decrease in bone mass.

It has been previously reported that body lipid percentage is positively correlated with reduced bone mass and that excess lipid causes a decrease in BMD and bone mineral content [22, 23]. It has also been demonstrated that excess adipose tissue affects bone metabolism and that obesity reduces osteoblast differentiation and bone formation while increasing adipogenesis. Obesity can also affect bone metabolism directly or indirectly through cytokines secreted by adipocytes such as leptin and adiponectin [24]. A study [25] found that leptin acts on the sympathetic nervous system via adrenergic β2 receptors to inhibit bone formation at the transcriptional level, and that lipocalin secreted by mature adipocytes can affect bone metabolism and decrease BMD by regulating the expression of osteoprotegerin [26]. The BMI of patients in the sarcopenia group in this study was significantly higher than that of the non-sarcopenia group, indicating the presence of higher lipid content in the sarcopenia group. In addition, when BMI ≥ 25, the negative correlation between lumbar BMD and sarcopenia was more pronounced, indicating the prevalence of increased lipid content, decreased bone density, and impaired muscle mass in the elderly. In conclusion, as a chronic disease of the elderly, OP has a slow and insidious pathogenesis and is known as a “silent killer” in academic circles. The link between “muscle–bone–lipid” is closely related to the pathogenesis of OP. The results of this study showed that the BMD of OP patients tended to decrease with age, and BMD is an important factor affecting lipid mass, muscle mass, and bone mineral content in the human body.

Until now, postmenopausal women have been the focus of most cohort and cross-sectional studies. The link between BMD and sarcopenia in young healthy people is poorly known. The findings are very applicable to the whole population since we chose a national comprehensive sample. Furthermore, due to our large sample size, we were able to perform a subgroup analysis of lumbar BMD and sarcopenia for people of different genders, ages, and BMI. The use of sensitivity analysis lowered the likelihood of false positives. However, it is critical to recognize the study’s limitations. Shortcomings of this study are that it shows that BMD is closely related to lipid, muscle, and other body components, and that skeletal muscle and lipid content are correlated with OP. However, the correlation between OP and sarcopenia has not been analyzed in depth. Second, due to the cross-sectional study design, we were unable to obtain a causal relationship between lumbar BMD and sarcopenia. More large sample prospective studies and basic mechanism studies are needed to understand the special mechanism of the association between BMD and sarcopenia. Third, there may be racial differences due to the NHANES database used in this study. Finally, other potential confounding factors that were not controlled for in this study were not ruled out as sources of bias. There are few studies on the link between “muscle–bone–lipid”, and this study is intended to investigate the interaction between the three components in the OP process and to provide a reference for further experimental studies.

## Conclusion

In this study, we used multiple linear regression models, smoothed curve fitting, and saturation effects analysis models to examine the relationship between lumbar BMD and sarcopenia in the US 20–59 population. In our analysis, we found not only a simple negative linear correlation between lumbar BMD and sarcopenia, but also saturation values that persisted across gender, age, and BMI subgroups. This work suggests that changes in lipid and muscle content with age, in addition to concomitant bone loss, imply the possibility of a strong “muscle–bone–lipid” link.

### Supplementary Information

**Additional file 1.** Figures S1 and S2; Table S1.

## Abbreviations

OP: Osteoporosis
NHANES: National Health and Nutrition Examination Survey
BMD: Bone mineral density
DXA: Dual-energy X-ray absorptiometry
BMI: Body mass index
ASM: Appendicular skeletal muscle mass
FNIH: Foundation for the National Institutes of Health
PIR: Poverty ratio
HDL-C: High-density lipoprotein cholesterol
LDL-C: Low-density lipoprotein cholesterol
