# Supplementary material for: Correlation between bone mineral density and sarcopenia in US adults: a population-based study
Source: J Orthop Surg Res. 2023 Aug 9;18:588. doi: 10.1186/s13018-023-04034-7 (PMC10410911; doi:10.1186/s13018-023-04034-7)
Supplement: Supplementary file 1 — Additional file 1. Figures S1 and S2; Table S1. [file 13018_2023_4034_MOESM1_ESM.docx]

**Supporting Information**

**Correlation between bone mineral density and sarcopenia in US adults: a population-based study**

Lulu Cheng^1,2*^ MD, Siyu Wang ^2^ MD

1. College of Acupuncture-Moxibustion and Tuina, Anhui University of Chinese Medicine Hefei 230012, China
2. Graduate School, Wuhan Sports University Wuhan 430079, China

E-mail:[Chenglulu958@163.com](mailto:Chenglulu958@163.com)

**Contents**

1. Supporting figures…………..…………………….………………………………….…..3

Figure S1 ………………………………………………………………………………....3

Figure S2 …………………………………………………………………………………3

Table S1 ………………………………………………………………………………….3

**1. Supporting figures**

| 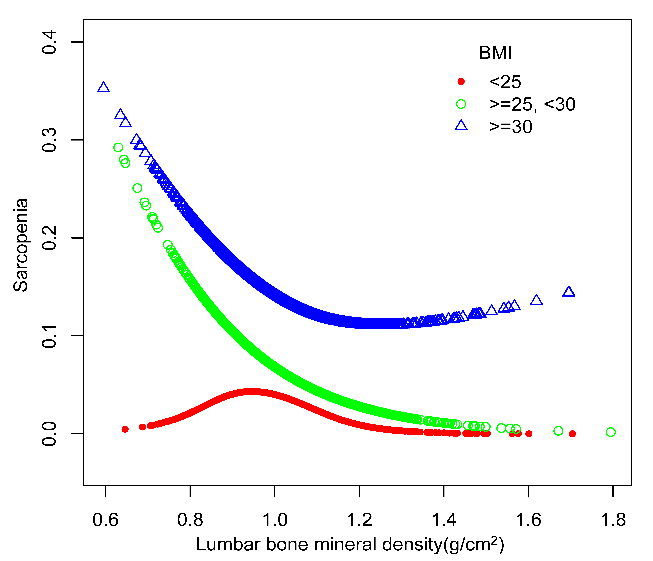 | 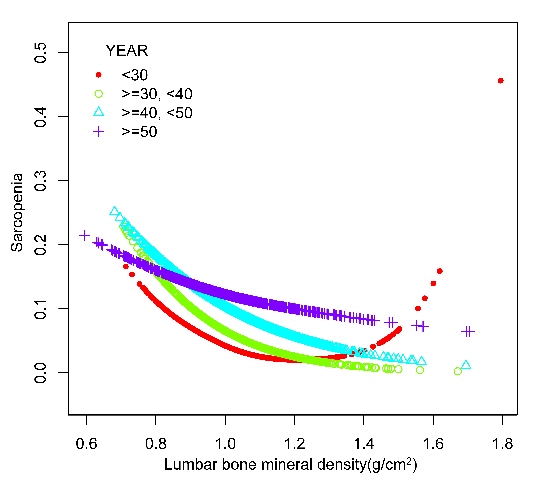 |
| --- | --- |
| **Figure S1. The association between lumbar BMD and sarcopenia was stratified by BMI.** Age, sex, race, education level, marital status, smoking status, alcohol status, diabetes status, hypertension status, creatinine, serum uric acid, glycohemoglobin, fasting glucose, HDL-C, total cholesterol, triglycerides, LDL-C, PIR, and albumin were adjusted. | **Figure S2. The association between lumbar BMD and sarcopenia stratified by age.** Sex, race, education level, marital status, smoking status, alcohol status, diabetes status, hypertension status, creatinine, serum uric acid, glycohemoglobin, fasting glucose, HDL-C, total cholesterol, triglycerides, LDL-C, PIR, and albumin were adjusted. |

**Table S1. Multivariate logistic regression models of sarcopenia**

|  | Model1 [OR (95% CI)] | Model2 [OR (95% CI)] | Model3 [OR (95% CI)] |
| --- | --- | --- | --- |
| Stratified by TC |  |  |  |
| <200 | 0.02(0.01,0.04) | 0.05(0.02,0.13) | 0.07(0.02,0.28) |
| ≥200 | 0.02(0.01,0.06) | 0.06(0.02,0.16) | 0.07(0.01,0.33) |
| Stratified by TG |  |  |  |
| <150 | 0.01(0.00,0.05) | 0.05(0.02,0.15) | 0.04(0.01,0.14) |
| ≥150 | 0.11(0.02,0.50) | 0.18(0.04,0.89) | 0.27(0.04,1.86) |
| Stratified by LDL-C |  |  |  |
| <130 | 0.02(0.01,0.06) | 0.05(0.01,0.17) | 0.05(0.01,0.17) |
| ≥130 | 0.07(0.02,0.28) | 0.18(0.04,0.79) | 0.08(0.02,0.45) |
| Stratified by HDL-C |  |  |  |
| <40 | 0.04(0.01,0.13) | 0.08(0.02,0.29) | 0.04(0.00,0.40) |
| ≥40 | 0.01(0.01,0.03) | 0.05(0.03,0.11) | 0.07(0.02,0.20) |
